# Research involvement among undergraduate health sciences students: a cross-sectional study

**DOI:** 10.1186/s12909-017-1025-x

**Published:** 2017-10-16

**Authors:** J. Bovijn, N. Kajee, T. M. Esterhuizen, S. C. Van Schalkwyk

**Affiliations:** 1 Faculty of Medicine and Health Sciences, Stellenbosch University, Tygerberg, Western Cape South Africa; 2 Centre for Evidence Based Health Care, Faculty of Medicine and Health Sciences, Stellenbosch University, Tygerberg, Western Cape South Africa; 3 Centre for Health Professions Education, Faculty of Medicine and Health Sciences, Stellenbosch University, Tygerberg, Western Cape South Africa

**Keywords:** Student research, Undergraduate research, Research participation, Research competence, Interprofessional education

## Abstract

**Background:**

The development of research capacity among undergraduates is an important intervention in countering the documented decrease in medical and health sciences researchers. The literature on undergraduate research generally emanates from smaller scale studies that have been conducted in high income countries, with a focus on medical students. This cross-sectional study was conducted in a Sub-Saharan country, included a population of medical and allied health professions (AHP) students, and aimed to improve our understanding of the factors influencing undergraduate student research.

**Methods:**

A questionnaire was distributed to all students enrolled in an undergraduate programme at the Faculty of Medicine and Health Sciences, Stellenbosch University, Cape Town, South Africa (including Medicine and four AHP programmes). Data was collected on a number of demographic characteristics and on 3 major outcome-themes: “voluntary research involvement”, “self-perceived research competence” and “future research participation”. Associations between characteristics and outcome themes were explored.

**Results:**

In total, 1815 students participated in the study (response rate 80.2%). Of all the demographic variables, discipline (AHP programmes vs. Medicine), male gender and prior undergraduate experience in a science degree were significantly associated with voluntary research involvement. Significantly higher levels of self-perceived research competence and greater interest in future research participation, were seen among participants from AHP programmes; males; and those with previous or current voluntary research involvement. Ethnicity and geographic background were not significantly associated with any of our outcomes.

**Conclusions:**

Our results offer important new evidence in support of the imperative to diversify the research work-force, in Sub-Saharan Africa and globally. Enhanced efforts aimed at achieving better academic representation in terms of gender, ethnicity, geographical and socio-economic backgrounds are strengthened by the findings of this study. Potential student researchers represent an important group amenable to further intervention. Further research may be required to explore the factors that determine the progression from interest to future participation in research.

**Electronic supplementary material:**

The online version of this article (10.1186/s12909-017-1025-x) contains supplementary material, which is available to authorized users.

## Background

A decline in the number of medical and health sciences researchers has been noted worldwide, including in the developing world [1–5]. Several different strategies have been proposed and implemented to counter this decline, with the development of research capacity amongst undergraduate health sciences students highlighted as an important intervention in this endeavour [6–9].

Actively participating in research as a student has been linked to higher research self-efficacy and competence levels [10–12], and growing literature supports the notion that research involvement among medical students may lead to increased research activity and outputs after graduation [13–15]. Although we know that student research may serve to strengthen pre-existing research interest, there is limited longitudinal evidence exploring how student research might otherwise lead to increased research participation after graduation [13, 14]. It is also not clear to what extent some demographic factors may influence student research or later research participation.

The need for diversification of the healthcare workforce has been noted globally, and particularly in developing countries [16–21]. In South Africa, medical schools have particularly focused on improving the gender, ethnic, linguistic, and geographic diversity of its student enrolment [20]. This is aimed at addressing historic inequalities, and also intends to decrease health inequities. It is, however not known what the effect of such diversification is on the research involvement and competence among health sciences students, and whether it may negatively influence scholarly participation in the future.

Even though we now have a reasonable amount of data on undergraduate student research, it generally emanates from smaller-scale studies, conducted primarily in high income countries, with limited data from lower and middle income countries. In addition, the work focuses on medical students and there remains a paucity of research looking at research involvement and attitudes among allied health professions (AHP) students. Allied health professionals remain underrepresented in clinical research, and this is thought to be, at least partially, due to a lack of skills, confidence and opportunities to participate in research [22]. In South Africa, the research component of AHP curricula may differ significantly between disciplines and institutions, and such components generally remain under-resourced. In the global context of an ever-growing burden of non-communicable disease and chronic conditions, often requiring preventative, curative and rehabilitative inputs over long periods of time, the role of the interdisciplinary team, and their participation in research, has become increasingly important [23–27]. Further, we would argue that with the increasing emphasis of the multidisciplinary team in health care, examining research attitudes across these disciplines, both individually and comparatively, is important.

In this article, we describe a study conducted at a medical and health sciences faculty in South Africa. We aimed to quantify voluntary research involvement in a group of medical and AHP students and to explore factors associated with such involvement. In addition, we sought to determine if voluntary research involvement and/or other demographic factors are associated with self-perceived research competence and attitude to future research participation. Knowing which factors encourage undergraduate student research across the different health professions can enable the implementation of meaningful educational interventions, and thereby facilitate its uptake.

## Methods

### Setting and participants

Our study was conducted at the Faculty of Medicine and Health Sciences (FMHS) at Stellenbosch University, Cape Town, South Africa. The faculty offers an undergraduate programme in Medicine and Surgery (MB,ChB – a 6 year programme); as well as four undergraduate AHP programmes: Bachelor of Science (BSc) in Dietetics (DT), Bachelor of Occupational therapy (OT), BSc in Physiotherapy (PT), and Bachelor of Speech, language and hearing therapy (SLHT), all 4 year programmes. Our target population included all undergraduate students registered for one of these programmes at the FMHS for the 2014 academic year, a total of 2263 students.

### Course context

At the FMHS, all undergraduate programmes incorporate small-scale curricular research projects (e.g. small surveys, clinical audits, etc.), typically during the more advanced years of the respective courses. Completion of such a curricular research project was explored in our study with a single item question. In addition, AHP students have to complete a compulsory final-year research thesis (also considered to be curricular research), whereas this is an optional component for medical students (and therefore regarded as voluntary in our study).

Given the historical inequalities in South African society, the quality of primary and secondary education varies widely across socioeconomic, ethnic and linguistic groups [18, 19] South African universities have developed a wide range of mechanisms to support the development of students from less privileged backgrounds. [20] The FMHS’ EDP-programmes lengthen the six-year MB,ChB programme to 7 years and the four-year AHP programmes to 5 years. This extends the first year over 2 years, with additional courses to prepare the student for the rest of the mainstream-programme.

### Questionnaire development

We adopted a cross-sectional design and utilised a 2.5-page, paper-based, self-administered questionnaire to collect our data (see Additional file 1). The questionnaire consisted of items across four main sections: a section on demographic characteristics and three sections exploring our major themes, i.e. “Voluntary research involvement”, “Self-perceived research competence” and “Future research participation”. Questions pertaining to “Voluntary research involvement” required a yes/no response (four items), whereas items relating to “Self-perceived research competence” and “Future research participation” were evaluated on 5-point Likert scales (eight and four items, respectively; Table 1). Other items included in the questionnaire were used for purposes other than the analyses described in this paper.

**Table 1:** Questionnaire themes and components

Theme and components	Measure	Analysis variable
Voluntary research involvement (4 questions: *I am/have*…)		
1. Currently working on a voluntary research project.	Yes / No	Binary (Yes to any of the four vs. no to all four).
2. Previously completed a voluntary research project.		
3. Previously presented your research at a congress or conference.		
4. Previously published your research in a medical journal.		
Self-perceived research competence (8 questions: *I am competent in*…)		
1. Searching the literature.	5-point Likert scales (strongly agree (assigned 5) to strongly disagree (assigned 1))	Continuous (Score out of 40 as a percentage).
2. Understanding and interpreting medical literature.		
3. Designing a research study.		
4. Conducting a research study.		
5. Analysing and interpreting data from a research study.		
6. Writing a medical paper/article.		
7. Knowing how to find a suitable research supervisor/mentor in my faculty.		
8. Knowing how to get involved with research and start my own research project.		
Future research participation (4 questions: *I am*…)		
1. Currently interested in pursuing a research project.	5-point Likert scales (strongly agree (assigned 5) to strongly disagree (assigned 1))	Continuous (Score out of 20 as a percentage).
2. Likely to get involved with research before graduating.		
3. Likely to pursue a career that involves conducting research.		
4. Likely to pursue a PhD in future.		

See Additional file 1 for sample questionnaire

Certain sections of the questionnaire were adapted from previously published studies [11, 28], and additional items were added to further characterise the demographic profile of our study population, the participants’ attitudes to future research involvement, as well as their previous and current degree of research involvement.

A pilot study was conducted prior to the data collection phase of the study to assess comprehensibility, and ease of completion. Pilot responses showed acceptable internal consistency reliability. Validity of the questionnaire was evaluated during a verbal debriefing session. No content changes were made following conclusion of the pilot phase.

### Data collection

Data collection took place from June to November 2014, covering all year-groups of all the programmes mentioned above (with the exception of 1st and 2nd year SLHT students, who attend classes on a different campus). Members of the research team (JB, NK) distributed the questionnaire to students in class or following formal tests or examinations. Completion was voluntary and anonymous, and typically took 5-10 min.

### Data management and analysis

Data entry was done in duplicate by two independent data-capturers and inconsistencies were validated against the original data-source. Questionnaires that were more than 50% incomplete, and those that were deemed invalid given inconsistency of responses (this was decided on the basis of 2 identical questions phrased in an inverse manner at different points in the questionnaire), were not included in the analysis.

Data analysis was conducted using SPSS, version 22 (IBM Corp © 2013). As per the pre-specified analysis plan, question items pertaining to “Self-perceived research competence” and “Future research participation” were grouped to create scores, whereas the “Voluntary research involvement” question-group was analysed as a single binary variable (see Table 1).

Factors associated with the 3 outcomes of interest were assessed using multiple logistic regression (using a backwards stepwise procedure based on likelihood ratio tests with a removal probability set at *p* = 0.1) in the case of the “Voluntary research involvement”-outcome, and multiple linear regression (using a backwards stepwise procedure based on F tests with a removal probability set at *p* = 0.1) in the case of the “Self-perceived research competence”- and “Future research participation”-outcomes. We entered all factors associated (*p* < 0.1) with the outcome on bivariate analysis. Missing categorical data for independent variables were coded as missing and included in the regression analyses but not reported in the final models. Missing data on items making up the defined scales were assumed to be missing at random, and imputed by using the item median for the sample to ensure that complete data were available for construction of scores. This method is efficient, and has been found to yield comparable results to multiple imputation methods [29].

The study was approved by the Stellenbosch University Health Research Ethics Committee and was carried out in accordance with the Declaration of Helsinki, including, but not limited to the anonymity of participants being guaranteed and the informed consent of participants being obtained.

## Results

### Demographic characteristics

Out of a total of 2263 registered students, 1815 valid responses were received (total response rate = 80.2%). Response rates varied between disciplines (MB,ChB 85.8%; DT 72.9%; OT 73.5%; PT 80.9%; SLHT 78.0%). This high response rate could be attributed to the fact that, although participation was voluntary, the request to complete the questionnaire came from a fellow student and recent graduate.

Demographic characteristics are summarised in Table 2. Median age for the whole group was 21 years (Range: 17-37 years).

**Table 2:** Demographic characteristics

Characteristic	*n*	% (out of 1815)
Sex		
Male	463	25.6
Female	1339	73.8
Not given	13	0.6
Discipline		
MB,ChB	1357	74.8
Dietetics	86	4.7
Occupational Therapy	136	7.5
Physiotherapy	190	10.5
Speech, language, hearing therapy	46	2.5
Average academic performance		
<60%	339	18.7
60-74%	1039	57.2
≥75%	387	21.3
Not given	50	2.8
EDP student (current or previous)^a^		
Yes	314	17.3
No	1414	77.9
Not given	87	4.8
Home language		
Afrikaans	625	34.4
English	829	45.7
Afrikaans and English	18	1.0
Other Southern-African language^b^	193	10.6
Other^c^	40	2.2
Not given	110	6.1
Previous years completed in other science degree		
No previous science degree	1446	79.7
≥1 year(s)	323	17.8
Not given	46	2.5
School-going environment^d^		
Urban	1472	81.1
Rural	336	18.5
Not given	7	0.4
Parents working in research-settings^e^		
None	1553	85.6
One	202	11.1
Both	43	2.4
Not given	17	0.9

^a^EDP (Extended Degree Programme): A faculty intervention which lengthens the six-year MB,ChB programme to 7 years and the 4 year programmes to 5 years. This extends the first year over 2 years, with additional courses to prepare the student for the remainder of the mainstream-programme

^b^Includes Xhosa, Zulu, Tswana, Northern Sotho/Sepedi, Venda, Tsonga, SiSwati, Ndebele, Zemba (Namibia), Shona (Zimbabwe)

^c^Includes German, French, Mandarin, Dutch, Burmese, Portugese, Igbo (Nigeria)

^d^Respondents were asked if they had attended primary or secondary school in a rural or urban environment (as two separate questions). If a respondent indicated at least one as rural (e.g. either primary or secondary or both), they would be categorised as “rural” for the purposes of analysis

^e^Respondents were asked if their parents worked in medical or non-medical research settings: tertiary hospitals, laboratory positions, university staff, etc. (either none, one, or both parents as options)

### Reliability

Grouped variables showed good internal consistency reliability with Cronbach’s alpha of 0.82 for the “Self-perceived research competence”-group and 0.80 for the “Future research participation”-group, and both variables approximated a normal distribution.

### General interest in research

Participants were asked how they generally felt about medical/clinical research; 1120 (61.7%) students indicated that they felt very positive or positive, 616 (33.9%) felt neutral, whilst 79 (4.4%) felt negative or very negative.

### Voluntary research involvement

Three-hundred participants (16.5%; 95% CI: (14.8 – 18.2%)) indicated that they had previously been or currently were involved in conducting some form of voluntary research. Of all the respondents, 1515 (83.5%) reported that they had no previous or current voluntary, extra-curricular research involvement (i.e. they answered no to all four questions – see Table 1). One-hundred-and 74 (9.6%) students had previously completed a voluntary research project, whilst 72 (4.0%) were currently busy with a voluntary project. In terms of outputs, 122 (6.7%) reported they had presented their research at a conference or congress, whilst 21 (1.2%) had published their research in a medical journal. Although curricular research may also have led to conference presentation or journal publication, the act of preparing such outputs was considered to be voluntary and extra-curricular.

Among those students with no previous or current voluntary involvement (*n* = 1515), 440 (29.0%) agreed or strongly agreed that they would be likely to get involved with research before graduating; whilst 255 (16.8%) agreed or strongly agreed that they were currently interested in becoming involved with research.

### Factors associated with voluntary research involvement

The final logistic regression model after 2 steps is shown in Table 3. Of all the demographic variables, discipline, gender and prior BSc experience were significantly associated with voluntary research involvement. The AHP programmes (with the exception of OT: OR = 1.41 (0.83-2.41), *p* = 0.206) were all significantly more likely to report voluntary research involvement than medical students. Male gender and those with prior BSc experience of 1 year or more, were also significantly more likely to be involved in voluntary research. Having previously completed a curricular research project, was also associated with voluntary research involvement.

**Table 3:** Multiple logistic regression analysis: Factors associated with voluntary research involvement (*n* = 1815)

Variables (Step 3)		OR	95% CI	*p*
	DT (vs. MB,ChB)	2.29	1.31 – 4.03	0.004
	OT (vs. MB,ChB)	1.41	0.83 - 2.41	0.206
	PT (vs. MB,ChB)	2.10	1.43 - 3.08	<0.001
	SLHT (vs. MB,ChB)	9.75	5.15 – 18.47	<0.001
	Male gender (vs. female)	1.99	1.48 - 2.67	<0.001
	Previous BSc experience (≥1 years vs. none)	1.60	1.17 - 2.20	0.003
	Previous curricular research project completed (yes vs. no)	3.27	2.46 – 4.34	<0.001

Variables entered on step 1: Discipline, Gender, BSc experience, Ethnicity, Age (*p* < 0.1 on bivariate analysis), previous curricular research project completed. *OR* Odds ratio, *CI* Confidence interval, DT Dietetics, *OT* Occupational therapy, *PT* Physiotherapy/Physical therapy, *SLHT* Speech, language and hearing therapy, *MB,ChB* Bachelor of Medicine, Bachelor of Surgery (MD/MBBS equivalent), *BSc* Bachelor of Science (undergraduate science degree)

### Self-perceived research competence

The mean “Self-perceived research competence”-score (*competence-score*; see Table 1 for details) for the whole study population was 56.9 (out of 100).

A multiple linear regression was carried out to ascertain the extent to which discipline, academic performance (self-reported), gender, home-language, previous BSc experience, and previous or current voluntary research involvement (all *p* < 0.1 on univariate analysis), are associated with *competence-score* (Table 4).

**Table 4:** Multiple linear regression model for “Self-perceived research competence”-score

Variables	B	SE	95% CI			Sig.
Intercept	59.12	0.97	57.22	–	61.02	<0.001
Discipline						
DT (vs. MB,ChB)	4.94	1.18	2.62	–	7.26	<0.001
OT (vs. MB,ChB)	3.10	0.97	1.21	–	5.00	<0.001
PT (vs. MB,ChB)	4.83	0.83	3.20	–	6.46	<0.001
SLHT (vs. MB,ChB)	11.12	1.62	7.94	–	14.30	<0.001
Academic performance						
< 60% (vs. ≥75%)	−3.04	0.79	−4.59	–	−1.50	<0.001
60-74% (vs. ≥75%)	−1.57	0.63	−2.80	–	−0.34	0.01
Gender						
Male (vs. female)	2.93	0.58	1.79	–	4.08	<0.001
Home Language						
Afrikaans (vs. English)	−0.65	0.56	−1.75	–	0.46	0.25
Other Southern-African language^a^ (vs. English)	1.57	0.85	−0.10	–	3.23	0.07
Afrikaans AND English^b^ (vs. English only)	5.38	2.51	0.47	–	10.29	0.03
Other^c^ (vs. English)	−0.96	1.70	−4.29	–	2.37	0.57
Previous BSc. experience						
≥ 1 years (vs. none)	1.50	0.66	0.22	–	2.79	0.02
Previous or current voluntary research involvement						
Yes (vs. none)	5.05	0.70	3.68	–	6.42	<0.001
Previous curricular research experience						
Yes (vs. none)	1.20	0.51	0.21	–	2.20	0.02

*B* Regression coefficient, *SE* Standard Error (of B), *DT* Dietetics, *OT* Occupational therapy, *PT* Physiotherapy/Physical therapy, *SLHT* Speech, language and hearing therapy,. *MB,ChB* Bachelor of Medicine, Bachelor of Surgery (MD/MBBS equivalent)

^a^Includes Xhosa, Zulu, Tswana, Northern Sotho/Sepedi, Venda, Tsonga, SiSwati, Ndebele, Zemba (Namibia), Shona (Zimbabwe)

^b^Participants that indicated a dual home-language (*n* = 18)

^c^Includes German, French, Mandarin, Dutch, Burmese, Portugese, Igbo (Nigeria)

The *competence-score* for students with voluntary research involvement was significantly higher than for those with no voluntary research involvement [mean difference 5.37 (95% CI 4.03 – 6.72; *p* < 0.001) (Table 4)].

Significantly higher *competence-scores* were seen among participants from the AHP programmes; those with better academic performance; those with prior BSc experience; males; and those with previous or current voluntary research involvement. In terms of language, the only significant association was found for those students reporting a dual home language of Afrikaans and English (*n*= 18; 0.01%). No other demographic variables (see Table 2) were found to be significantly associated with this outcome.

### Future research participation

The mean “Future research participation”-score (*future participation-score*; see Table 1 for details) for the whole study population was 59.4 (out of 100).

A multiple linear regression was carried out to ascertain the extent to which discipline, gender, home-language, parental employment in a research-setting, and previous/current voluntary research involvement (all *p* < 0.1 on univariate analysis), are associated with *future participation-score* (Table 5).

**Table 5:** Multiple linear regression model for “Future research participation”-score

Variables	B	SE	95% CI			Sig.
Intercept	62.20	1.42	59.42	–	64.98	<0.001
Discipline						
DT (vs. MB,ChB)	3.01	1.62	−0.16	–	6.19	0.06
OT (vs. MB,ChB)	−0.49	1.32	−3.08	–	2.10	0.71
PT (vs. MB,ChB)	2.72	1.13	0.51	–	4.94	0.02
SLHT (vs. MB,ChB)	5.62	2.22	1.28	–	9.97	0.01
Gender						
Male (vs. female)	2.74	0.80	1.17	–	4.31	0.001
Home Language						
Afrikaans (vs. English)	−2.30	0.76	−3.80	–	−0.80	0.003
Other Southern-African language^a^ (vs. English)	6.79	1.16	4.52	–	9.07	<0.001
Afrikaans AND English^b^ (vs. English only)	8.12	3.43	1.41	–	14.84	0.02
Other^c^ (vs. English)	3.45	2.33	−1.11	–	8.01	0.14
Parents working in research-settings						
Only one parent (vs. neither)	1.63	1.08	−0.50	–	3.74	0.13
Both parents (vs. neither)	6.42	2.22	2.06	–	10.78	0.004
Previous or current voluntary research involvement						
None (vs. present)	4.01	0.95	2.15	–	5.88	<0.001

*B* Regression coefficient, *SE* Standard Error (of B), *DT* Dietetics, *OT* Occupational therapy, *PT* Physiotherapy/Physical therapy, *SLHT* Speech, language and hearing therapy, *MB, ChB* Bachelor of Medicine, Bachelor of Surgery (MD/MBBS equivalent)

^a^Includes Xhosa, Zulu, Tswana, Northern Sotho/Sepedi, Venda, Tsonga, SiSwati, Ndebele, Zemba (Namibia), Shona (Zimbabwe)

^b^Participants (*n* = 18) that indicated a dual home-language

^c^Includes German, French, Mandarin, Dutch, Burmese, Portugese, Igbo (Nigeria)

The mean *future participation-score* for students with voluntary research involvement was significantly higher than for those with no voluntary research involvement [mean difference 3.82 (95% CI 1.99 – 5.64; *p* < 0.001) (Table 5)].

Significantly higher *future participation-scores* were seen among participants from the PT and SLHT programmes (when compared to MB,ChB); males; those with previous or current voluntary research involvement, and students with both parents working in research-settings. Students reporting a dual home language of Afrikaans and English, as well as those speaking a native Southern-African language (excluding Afrikaans or English) as home-language, were significantly more likely to report higher *future participation-scores.* No other demographic variables (including previous curricular research experience) were found to be significantly associated with this outcome.

## Discussion

Our study represents a population of medical and allied health professions (AHP) undergraduates from a sub-Saharan country. To date, it is one of the largest single-site studies in the field of health sciences student research, and is unique in its inclusion of AHP students. A number of previously established findings have been found to concur with our population, whilst important new evidence has also been established.

### Allied health professions students

AHP students were generally more likely to be involved in voluntary research when compared to medical students (with the exception of OT students). These students were also more likely to report higher self-perceived research competence levels, as well as a greater tendency to indicate an interest in participating in research in the future (the latter outcome with the exception of DT and OT students). These findings show that AHP students are important current and potential academic contributors and emphasise the value of formal and structured exposure to the practice of research at undergraduate level (since AHP students have to complete a compulsory final-year research thesis).

The role of educational culture in the various disciplines, and perceptions of future research opportunities were not explored in this study. It is plausible that medical students have a perception of having more research opportunities after graduation, and may therefore feel less inclined to do research before graduating. Further research into the role of the curriculum and other factors would be required to better understand why AHP students in our faculty are more likely to participate in voluntary research. We do believe these findings support the inclusion of AHP students in interventions aimed at increasing research involvement among medical students.

### Language, ethnicity and curricular extension

Our study found that EDP-programme students did not significantly differ in terms of our three study outcomes, when compared to non-EDP (i.e. traditional/mainstream) students. Ethnicity was also not significantly associated with any of our three main outcomes. Home-language (a diverse demographic variable in the South African context – see Table 2) was not significantly associated with voluntary research involvement or self-perceived competence; however, with regards to future research participation, home-language speakers of native Southern African languages (i.e. excluding English or Afrikaans home-language speakers), had significantly higher scores. Whilst the latter finding is an interesting result, it is not clear from this study why this might be the case. Further study would be required to gain a better understanding of how and why attitudes towards research might differ between ethnic, linguistic and cultural groups. Altogether, our findings represent important new evidence in support of policies aimed at addressing historical inequalities. As South Africa aims to increase training of a more diverse health care workforce [18, 20] – including increasing health research capacity development in underrepresented populations [7] – our results significantly strengthen the rationale behind these interventions. In addition, the need for greater diversification of the health sciences research workforce in developed countries has also been shown, emphasising the global relevance of our findings [21].

### Geographic origin

Exploring the impact of rural versus urban background on research involvement is also important in the South African and Sub-Saharan context, as a large proportion of the population resides in rural areas, while rural populations are generally underserved and have inferior access to healthcare [18, 19, 30]. Previous studies have consistently shown that students from rural backgrounds are more likely to practice in a rural setting [30–32]. Our findings show that rural students did not significantly differ in terms of our three study outcomes, when compared to students from an urban background. This is encouraging in light of the imperative to increase rural student recruitment.

### Prior experience in a formal science degree

South Africa does not require a pre-medical degree, and the majority of students enter a programme in medicine or AHP directly from high school at age 18. Intercalated programmes are uncommon [33]. A small minority of students complete a year in a formal science degree (BSc), while even fewer complete a full 3 to 4 year BSc programme, prior to entering medicine or AHP. Students that had previously completed at least 1 year in a BSc degree (prior to their current degree) were more likely to participate in voluntary research, and more likely to have higher self-perceived research competence. The effect of BSc experience in our study, may be likened to the positive effect of an additional, formal science-degree found in other studies [13, 34]. We speculate that a major reason for this may be that BSc programmes tend to focus on research and science as core curricular components, whereas medicine and AHP programmes tend to offer more practice-based curricula. Interestingly, students who reported having both their parents working in a research setting (medical or non-medical), were significantly more likely to report positive attitudes toward future research participation. This may also be related to prior exposure to scientific and research-related principles.

### Gender

Male gender was associated with a significantly increased voluntary involvement rate, higher self-perceived competence scores, and more positive attitudes toward future participation. This is a concerning finding, given that nearly three-quarters (73.8%) of the total FMHS undergraduate population (90.0% for AHP programmes) is female. This finding is consistent with established data, and the proposed reasons for this “gender-gap” are complex [13, 35, 36]. A lack of female mentor-figures, perpetuated sexism in the research environment (including inequality in the allocation of resources, remuneration, etc.), established concepts of traditional gender-roles, and differences in career preference (e.g. choosing clinical practice over academia), have been shown to explain at least part of this imbalance [35]. Interventions aimed at reversing this trend should enjoy priority among educationalists.

### Potential student researchers

Previous or current voluntary research experience was significantly associated with higher levels of self-perceived research competence, as well as interest in future research participation. This finding further emphasises the importance of supporting research development at the medical student level, and expands this to include AHP students.

Finally, this study has detected an important group of students – the so-called “potential student researchers”. These are students with no previous or current voluntary research involvement, but with an inclination for conducting research as students. In our study between 16 and 29% of students with no previous or current voluntary involvement, could be seen as potential student-researchers (see results section *Voluntary research involvement*). Future research and initiatives should focus specifically on the identification of this group of students and on finding ways of nurturing their research interest and supporting their academic development.

### Limitations of the study

In determining the prevalence of voluntary research involvement, participants were asked if they had previously published their research in a “medical journal”. This may have limited some participants from reporting peer-reviewed publication, as the term “medical journal” may have been interpreted as being restrictive. This may have led to a falsely low representation of the number of students who had published their research. Use of the term “health sciences journal” may have been more appropriate.

Location and timing of data collection may have influenced responses, e.g. some students were required to answer the questionnaire after completing a formal assessment, whereas other students completed it in class. This was required to ensure the appropriate response rates.

## Conclusions

AHP students have been shown to be important academic contributors, and these students should enjoy the same attention and interventions with regards to their academic development, as do medical students. The impact of having a structured, curricular research project is potentially an important part of fuelling interest in research.

Our findings offer important new evidence in support of the imperative to diversify the research work-force in Sub-Saharan Africa and globally. Enhanced efforts aimed at achieving better academic representation in terms of gender, ethnicity, geographical and socio-economic backgrounds are strengthened by our study.

Potential student researchers represent an important group amenable to further intervention. Further research may be required to explore the factors that determine the progression from interest in research to participation in research.

Internationally, health professions education is being entreated to shift its thinking and refine its approaches towards teaching and training. New foci include notions of transformative learning and interdependence in education which are set against a backdrop of graduating emergent leaders who have a strong sense of social accountability [37]. These graduates will, however, have an equal responsibility to shape the science of health care going forward. Knowing the factors that might influence undergraduate health sciences students to take on the mantle of becoming the next generation of researchers can be of considerable value for those responsible in designing undergraduate curricula.

## Additional file

Additional file 1: Questionnaire used in the study. (PDF 399 kb)

## Abbreviations

AHP: Allied health professions
BSc: Bachelor of Science
DT: BSc in dietetics
FMHS: Faculty of Medicine and Health Sciences
MB,ChB: Bachelor of medicine and surgery (medical degree)
OT: Bachelor of occupational therapy
PT: BSc in Physiotherapy
SLHT: Bachelor of Speech, language and hearing therapy (SLHT)
SU: Stellenbosch University
