# Supplementary material for: Research involvement among undergraduate health sciences students: a cross-sectional study
Source: BMC Med Educ. 2017 Oct 16;17:186. doi: 10.1186/s12909-017-1025-x (PMC5644181; doi:10.1186/s12909-017-1025-x)
Supplement: Additional file 1: — Questionnaire used in the study. (PDF 399 kb) [file 12909_2017_1025_MOESM1_ESM.pdf]

Office Use

A cross-sectional study characterising interest in research and scholarly activity among undergraduate medical and health science students.

The aim of this study is to explore the interest in research and scholarly activity among undergraduate medical and health sciences students. **Participation is completely anonymous.**

For the respondent: I understand that by completing this questionnaire I consent to participating in this part of the study. I understand the purpose, scope and potential outcomes of the study. I understand that this research is voluntary and that I am not required to participate.

Please indicate choice with a 'X' or fill in where indicated.

**Current Course:**	MB, ChB	Dietetics	Physiotherapy	Occ. Therapy	SLHT	
Are you a **current** or **previous EDP student?**	Yes, Current	Yes, Previous	No			
**Year** of study:	I	II	III	IV	V	VI
**Average academic performance** during course of your studies:	<50%	50-59%	60-74%	75-79%	80%+	
**Gender**:	M	F				
	**Age**:					
**Ethnicity**:	Caucasian/white	Black	Coloured	Indian	Other	
Home **language**/mother-tongue:	Afrikaans	English	Xhosa	Other:		
Previous **years of study completed** in a **science** degree (e.g. BSc):	0	1	2	3	4 or more	
Number of **parents** who are **health care professionals**:	0	1	2			
Number of **parents** who work in **medical or non-medical research** settings: (e.g. tertiary hospitals, laboratory positions, university staff, etc.)	0	1	2			
Did you attend **primary school** in a rural or urban setting?	Rural	Urban				
Did you attend **high school** in a rural or urban setting?	Rural	Urban				

Please describe in **one sentence**, what you understand by the term "research"

How do you **generally feel** about medical/clinical research?	Very Negative	Negative	Neutral	Positive	Very Positive

Have you previously completed a research project **as part of the curriculum** (e.g. family medicine rotation)?	Yes	No
Are you **currently working** on a **voluntary research project**? (I.e. **not** as part of the formal curriculum, but including final-year "Skripsies")	Yes	No
Have you previously **completed** a **voluntary research project** (I.e. **not** as part of the formal curriculum, but including final-year "Skripsies")?	Yes	No
Have you previously **presented** your research at a conference or congress?	Yes	No
Have you previously **published** your research in a medical journal?	Yes	No
Have you ever attended a presentation at the **Faculty's Annual Academic Day**?	Yes	No

How often do you refer to **medical literature** (for instance, reading a journal article)?	Daily	Weekly	Monthly	Almost never
Are you aware of any **research opportunities available** at our Faculty?	Very aware	Somewhat aware	Mostly unaware	Totally unaware

If you are aware, please give an example of research projects that you are aware of?

Please rate the statements below, with a "x":	Strongly agree	Agree	Neutral	Disagree	Strongly disagree
I am **currently interested** in pursuing a research project.					
I am **likely to get involved** with research before graduating.					
I am likely to **pursue a career** that involves conducting research.					
I am likely to pursue a **PhD** in future.					
I am likely to **never get involved with any research** activities.					
I am likely to **temporarily** move overseas for **academic** reasons (e.g. specialising, research, etc).					
I am likely to **permanently** move overseas for **academic** reasons (e.g. specialising, research, etc).					

Please rate the statements below, with a "x":	Strongly agree	Agree	Neutral	Disagree	Strongly disagree
Research projects should be made **compulsory** for all medical/health science students.					
Participating in research will be of value in my job as a **clinician**.					
Participating in research will be necessary to **achieve my career goals**, e.g. becoming a specialist.					
Participating in research is an important part of my medical education.					
Research is too **challenging**.					
Research is **interesting**.					

I am likely to specialise.	Yes	No	Don't know
If YES, please state field of likely specialisation			

Please rate the statements below, with a "x":	Strongly agree	Agree	Neutral	Disagree	Strongly disagree
There is **adequate time** during my studies to pursue research.					
There should be **time set aside** in the curriculum for pursuing research interests.					
There is **adequate training** for undergraduates in **research methods**.					
There is **adequate training** for undergraduates in **finding and reviewing scientific literature**.					
It is difficult as an undergraduate to attain adequate research **funding**.					
I know how to find a suitable research **supervisor**/mentor in my faculty.					
I know how to get involved with research and **start** my own research project.					
It is difficult to **present** research (e.g. at a conference) as an undergraduate student.					
It is difficult to **publish** research (e.g. in a journal) as an undergraduate student.					

I have read through the **Research Toolkit**.	Yes	No	Don't know what the Research Toolkit is

<u>I am **competent** in the following:</u>	Strongly agree	Agree	Neutral	Disagree	Strongly disagree
**Searching** the literature					
**Understanding and interpreting** medical literature					
**Designing** a research study					
**Conducting** a research study					
**Analysing and interpreting** data from a research study					
**Writing** a medical paper/article					
If you have previously completed a formal research project, please share some of your experiences:					
Please give us your **suggestions for making research more accessible to students**:					
This questionnaire was **easy to understand and complete**:	**Strongly agree**	**Agree**	**Neutral**	**Disagree**	**Strongly disagree**

Thank you for participating. The results of the study will be made known to all students when all data has been collected and analysed.

For queries or specific feedback, please contact the investigators via email: **tygerbergresearch@gmail.com**
